# Palynological and X-ray fluorescence (XRF) data of Carnian (Late Triassic) formations from western Hungary

**DOI:** 10.1016/j.dib.2019.103858

**Published:** 2019-03-20

**Authors:** Viktória Baranyi, Ágnes Rostási, Béla Raucsik, Wolfram Michael Kürschner

**Affiliations:** a Department of Geosciences, University of Oslo, P.O. Box 1047, Blindern, 0316 Oslo, Norway; b Department of Earth and Environmental Sciences, University of Pannonia, P.O.Box 158, H-8201 Veszprém, Hungary; c Department of Mineralogy and Geochemistry, University of Szeged, Egyetem Utca 2–6, H-6722 Szeged, Hungary

## Abstract

The data presented in this article are related to the research article “Palynology and weathering proxies reveal climatic fluctuations during the Carnian Pluvial Episode (CPE) (Late Triassic) from marine successions in the Transdanubian Range (western Hungary)” (Baranyi et al., 2019). Palynological and palynofacies counts and mineralogical data are presented that build the core for the palaeoenvironmental and palaeoclimatic interpretation discussed in the original research article. Other component of this data article is the description of the applied laboratory and analytical techniques. We also supply microscopic images of the identified pollen and spores and a list of all identified palynomorphs.

**Table:** Specifications table

Subject area	*Geology*
More specific subject area	*Palynology and inorganic geochemistry, palaeoclimate analysis*
Type of data	*Tables with palynological counts and XRF data, microscopy images, texts*
How data was acquired	*Core sample collection, microscope survey for palynology and palynofacies analysis and XRF*
Data format	*Raw data collection (MS Excel Sheets), Tables in MS Word format, microscope images, description of analytical and statistical techniques*
Experimental factors	*Palynological preparation techniques and XRF analysis*
Experimental features	*Standard procedures of laboratory preparation techniques and light microscopy analysis*
Data source location	*Hungary*
Data accessibility	*The data are available with this article.*
Related research article	*Baranyi* et al. *(2019)**[1]*

**Table:** 

**Value of the data** • Data provide the basis of the palaeoclimatic interpretation across the Carnian Pluvial Episode (CPE) • Data complement other paleontological and geochemical studies across the CPE • High resolution quantitative palynological data from the Carnian of the Transdanubian Range (western Hungary) • Mineralogical data are applied to determine weathering proxies • The presented data could motivate the integration of palynology and mineralogical data in the future in order to understand the CPE more effectively

## 1 Data

**Fig. 1:**
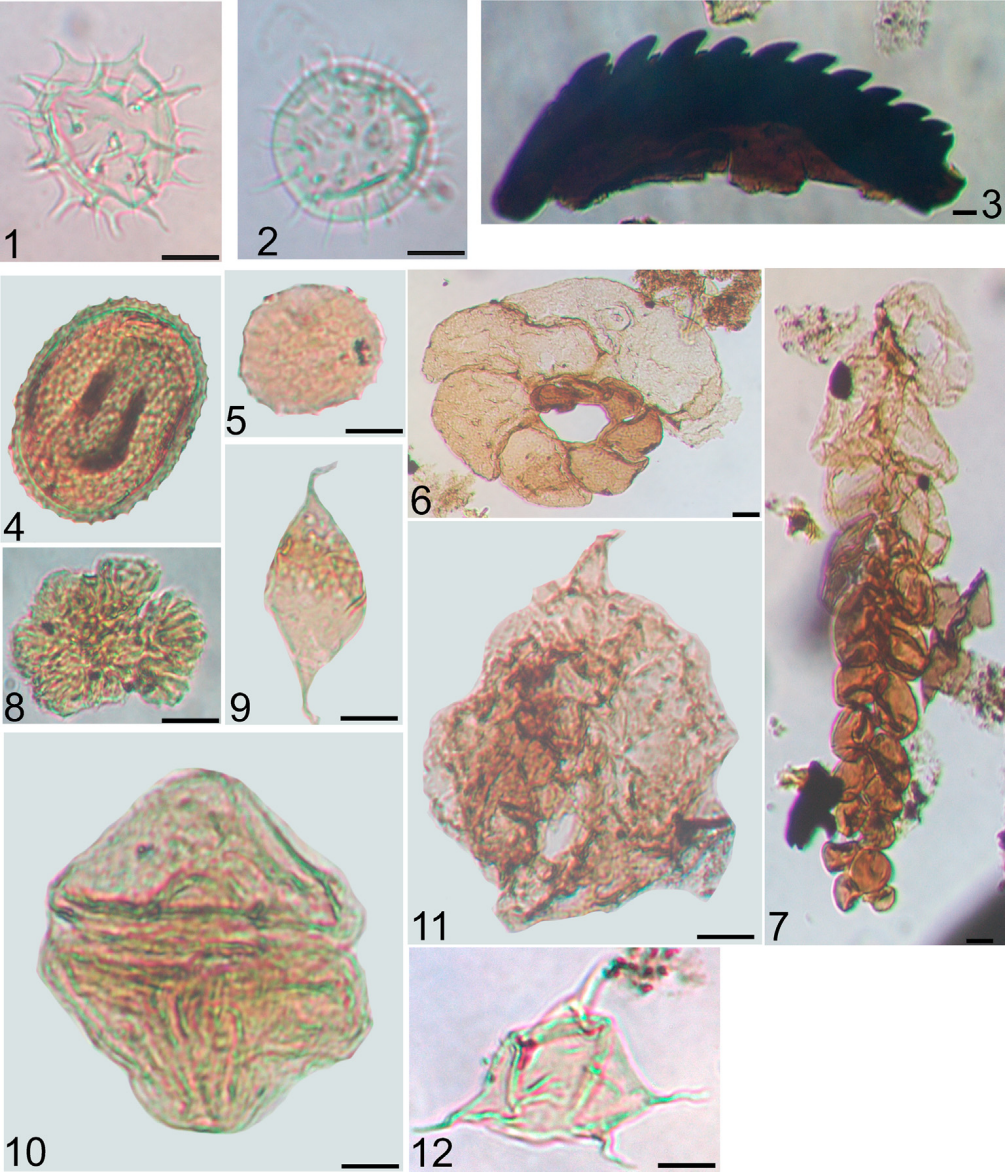
Aquatic palynomorphs from the Veszprém Formation, with the indication of sample code, sample code refers to the depth in meters; Met refers to samples from borehole Mencshely-1, V from Veszprém-1. Scale 10 μm. 1. *Micrhystridium* sp. 2. V-1/578; 2. *Baltisphaeridium* sp. V-1/573; 3. Scolecodont V-1/532; 4. *Tasmanites* sp. Met-1/122.9; 5. *Cymatiosphaera* sp. V-1/343; 6. Foraminiferal test lining Met-1/150; 7. Foraminiferal test lining V-1/485; 8. *Botryococcus braunii* Met-1/81; 9. *Leiofusa* sp. V-1/549; 10. *Heibergella* sp. Met-1/325; 11. Dinocyst indet. Met-1/122.9; 12. *Veryhachium* sp. Met-1/69.8.

**Fig. 2:**
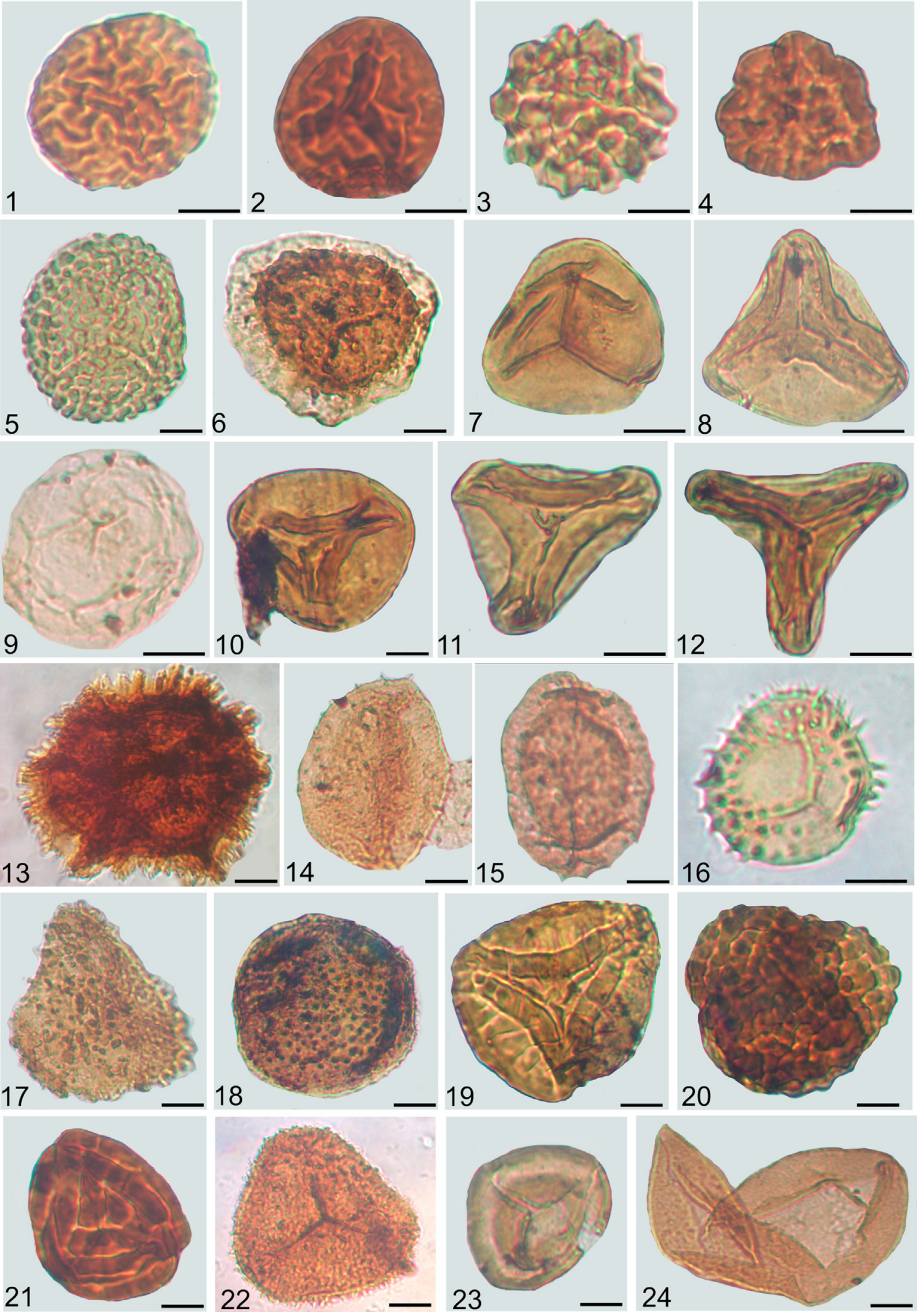
Spores from the Veszprém Formation and Csákberény Formation, with the indication of sample code and slide number, sample code refers to the depth in meters; Met refers to samples from borehole Mencshely-1, V-1 from Veszprém-1, Zs from Zsámbék-14. Scale 10 μm. 1. *Lycopodiacidites kuepperi* V-1 334.6/1; 2. *Camarazonosporites rudis* V-1 343/2; 3. *Gibeosporites lativerrucosus* V-1 335/1; 4. *Uvaesporites gadensis* V-1 343/2; 5. *Verrucosisporites morulae* V-1 350/1; 6. *Kraeuselisporites cooksonae* V-1 532/1; 7. *Deltoidospora* sp. Met-1 299.5/1; 8. *Dictyophillidites harrisii* V-1 491–492/1; 9. *Calamospora tener* V-1 578/1; 10. *Laevigatisporites robostus* Met-1 199.4/1; 11. *Paraconcavisporites lunzensis* Met-1 87/1; 12. *Concavisporites toralis* Met-1 135/1; 13. *Reticulatisporites dolomiticus* V-1 334.6/1; 14. *Aratrisporites palettae* V-1 573/2; 15. *Aratrisporites scabratus* V-1 343/2; 16. *Anapiculatisporites telephorus* Met-1 177.4/1; 17. *Neoraistrickia taylorii* Met-1 252/1; 18. *Porcellispora longdonensis* Met-1 135/1; 19. *Kyrtomisporits erveii* Zs 329.7/1; 20. *Converrucosisporites tumolosus* tetrad Zs 329.7/1; 21. *Striatella seebergensis* Met-1 91/1; 22. *Conbaculatisporites mesozoicus* V-1 343/1; 23. *Rogalskaisporites* sp. V-1 334.6/1; 24. *Todisporites major* V-1 493/2.

**Fig. 3:**
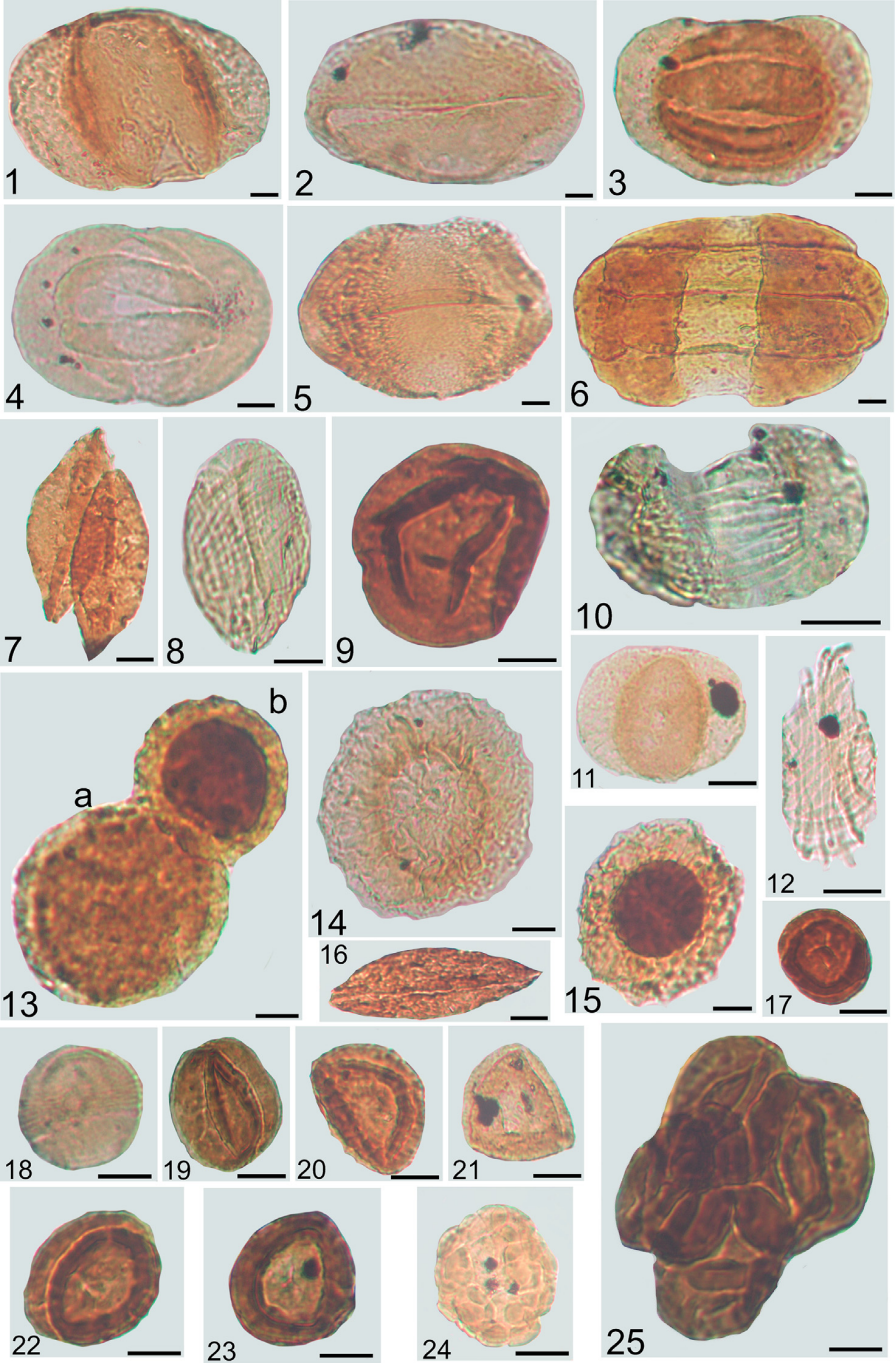
Pollen grains from the Veszprém Formation and Csákberény Formation, with the indication of sample code and slide number, sample code refers to the depth in meters; scale 10 μm, Met refers to samples from borehole Mencshely-1, V-1 from Veszprém-1, Zs from Zsámbék-14. 1. *Alisporites aequalis* Met-1 122.9/1; 2. *Ovalipollis ovalis* V-1 343/2; 3. *Lunatisporites acutus* V-1 343/1, 4. *Lueckisporites singhii* V-1 573/1; 5. *Staurosaccites quadrifidus* V-1 343/2; 6. *Infernopollenites sulcatus* Met-1 101.4/1; 7. *Cycadopites* sp. V-1 493/1; 8*. Lagenella martinii* Met-1 299.5/1; 9*. Aulisporites astigmosus* V-1 335/1; 10. *Striatoabietites aytugii* Zs 373.2/1; 11. *Triadispora crassa* V-1 573/1; 12. *Equisetosporites chinleana* V-1 506/1; 13. a) *Enzonalasporites vigens* b) *Enzonalasporites tenuis* Met-1 252/1; 14. *Patinasporites densus* V-1 343/2; 15. *Patinasporites explanatus* V-1 343/2; 16. *Cycadopites* sp. V-1 493/2; 17. *Partitisporites tenebrosus* Met-1 122.9/1; 18. *Partitisporites maljawkinae* Met-1 81/2; 19. *Partitisporites tenebrosus* V-1 491–492/1; 20. *Duplicisporites mancus* Met-1 122.9/1; 21. *Duplicisporites granulatus* Met-1 122.9/1; 22. *Duplicisporites continuus* Met-1 252/1; 23. *Duplicisporites continuus* V-1 491–492/1; 24. *Camerosporites secatus* V-1 335/1; 25. *Partitisporites tenebrosus* tetrad V-1 343/2.

This article describes the palynological and mineralogical data of Carnian formations (Late Triassic) from the Transdanubian Range (western Hungary). The palynological content includes the raw palynological and palynofacies counts from the 83 studied samples (Supplementary S1eS3). The article contains the list of all identified palynomorphs (Supplementary S4) and Fig. 1, Fig. 2, Fig. 3 document the most significant spore-pollen and aquatic palynomorph types. Mineralogical data and the calculated weathering indices are shown in Supplementary S6. In addition, the article presents the applied palynofacies terminology (Table 1) and the literature compilation that was used in the palaeoecological interpretation of the spore-pollen assemblages. (Table 2).

**Table 1:** Summary of palynofacies terminology. The terminology is used from Oboh-Ikuenobe and de Villiers ([6]).

Sedimentary organic particles (SOM)	Description
Amorphous organic matter (AOM)	Structureless, irregularly shaped, fluffy yellowish-brown to black masses that can be derived from the degradation of terrestrial or marine organic matter.
Charcoal/black debris	Totally opaque particles with variable shape and size. They are derived from highly oxidised wood or other plant debris.
Plant tissues (Structured translucent plant debris)	Structured transparent particles with yellow-green to brown colour. They may be derived from degraded plant tissues or wood. They are of various shape and size including lath-shaped and equidimensional particles.
Cuticles	Epidermal cells of higher plants' leaves and stems, often pale yellow to pale brown in colour. They typically possess rounded or polygonally-shaped cells.
Wood fragments	Structured lath-shaped or usually blocky particles, varying from pale yellow to brown in colour, often with cellular structure.
Resin	Translucent, colourless or yellow to red, globular particles, angular fragments or bubbly masses, produced by higher land plants.
Spores	Male reproductive organs of bryophytes and pteridophytes
Pollen grains	Male reproductive organs of the seed plants
Freshwater algae	*Botryococcus*, *Schizosporis*
Marine palynomorphs	Dinocysts, acritarchs, prasinophytes, scolecodonts and chitinous inner linings of the foraminifera

**Table 2:** Botanical affinity, proposed habitat and ecological affinity of the identified palynomorphs. Botanical affinities from [9]. Ecology from [7], [8], [9]. SEGs from [8].

Taxa	Botanical affinity	Ecology	SEGs
*Anapiculatisporites telephorus*	lycopsid?	hygrophyte	wet lowland
*Aratrisporites* spp.	lycopsid	hygrophyte	coastal
*Camarazonosporites rudis*	lycopsid	hygrophyte	river
*Calamospora tener*	Equisetales	hygrophyte	river
*Baculatisporites* sp.	Filicopsida	hygrophyte	wet lowland
*Conbaculatisporites mesozoicus*	Dipteridaceae	hygrophyte	river
*Concavisporites toralis*	Matoniaceae	hygrophyte	wet lowland
*Converrucosisporites tumolosus*	Dicksoniaceae	hygrophyte	wet lowland
*Cyclogranisporites sp.*	Osmundaceae	hygrophyte	river
*Deltoidospora* sp.	Filicales	hygrophyte	dry lowland
*Dictyophyllidites harrisii*	Filicales	hygrophyte	dry lowland
*Gibeosporites lativerrucosus*	Filicopsida	hygrophyte	wet lowland
*Gordonispora fossulata*	bryophyte	hygrophyte	river
*Kraeuselisporites cooksonae*	lycopsid	hygrophyte	coastal
*Kyrtomisporis erveii*	fern	hygrophyte	dry lowland
*Laevigatisporites robostus*	Filicales?	hygrophyte	dry lowland
*Leschikisporis aduncus*	Marrattiales	hygrophyte	coastal
*Lycopodiacidites kuepperi*	lycopsids	hygrophyte	river
*Neoraistrickia taylorii*	lycopsid	hygrophyte	river
*Osmundacidites wellmanni*	Osmundaceae	hygrophyte	wet lowland
*Paraconcavisporites lunzensis*	Filicales	hygrophyte	dry lowland
*Porcellispora longdonensis*	liverwort	hygrophyte	river
*Reticulatisporites dolomiticus*	fern, lycopsid	hygrophyte	coastal
*Striatella seebergensis*	Filicopsida	hygrophyte	coastal
*Todisporites* spp.	Osmundaceae	hygrophyte	river
*Uvaesporites gadensis*	Selaginellales	hygrophyte	river
*Verrucosisporites morulae*	Filicales	hygrophyte	wet lowland
*Zebrasporites* sp.	Filicales	hygrophyte	wet lowland
*Alisporites* spp.	seed fern	hygrophyte?	dry lowland
*Brachysaccus neomundanus*	conifer	xerophyte	dry lowland?
*Ellipsovelatisporites plicatus*	conifer	xerophyte	hinterland
*Infernopollenites* spp.	conifer	xerophyte	hinterland
*Lueckisporites singhii*	Majonicaceae		hinterland
*Lunatisporites acutus*	Voltziaceae	xerophyte	hinterland
*Microcachrydites doubingeri*	Podocarpaceae	xerophyte	hinterland
*Minutosaccus crenulatus*	Voltziaceae	xerophyte	hinterland
*Ovalipollis* spp.	Voltziaceae	xerophyte	hinterland
*Parillinites sp.*	conifer?	xerophyte	hinterland
*Pityosporites/Protodiploxypinus*	conifer/seed fern	xerophyte	hinterland
*Platysaccus queenslandi*	Podocarpaceae	xerophyte	coastal
*Staurosaccites quadrifidus*	unknown	xerophyte?	hinterland
*Striatoabietites aytugii*	seed fern	xerophyte	hinterland
*Sulcatisporites krauseli*	conifer?	xerophyte	hinterland
*Triadispora* spp.	Voltziaceae	xerophyte	hinterland
*Enzonalasporites* spp.	Majonicaceae	xerophyte	hinterland
*Patinasporites* spp.	Majonicaceae	xerophyte	hinterland
*Pseudoenzonalasporites summus*	Majonicaceae	xerophyte	hinterland
*Vallasporites ignacii*	Majonicaceae	xerophyte	hinterland
*Camerosporites secatus*	Cheirolepidiaceae	xerophyte	hinterland
*Duplicisporites* spp.	Cheirolepidiaceae	xerophyte	hinterland
*Partitisporites* spp.	Cheirolepidiaceae	xerophyte	hinterland
*Praecirculina granifer*	Cheirolepidiaceae	xerophyte	hinterland
*Laricoidites* sp.	Araucariaceae	xerophyte	coastal
*Aulisporites astigmosus*	Bennettitales	hygrophyte	dry lowland
*Brodispora striata*	?	hygrophyte	NA
*Cycadopites* sp.	Cycadales	hygrophyte	dry lowland
*Equisetosporites chinleana*	Gnetales	xerophyte	dry lowland
*Lagenella martinii*	?	?	NA
*Retisulcites* sp.	?	?	NA

## 2 Experimental design, materials and methods

### 2.1 Materials

Palynology and mineralogical analysis are performed on the same samples as in [2], [3]. For palynological and palynofacies analysis 83 samples were taken from three boreholes in the Transdanubian Range (western Hungary). In the Balaton Highland-Bakony Mountains area two borehole sections were studied. The Veszprém–1 (V–1 borehole; N 47°112, E 17°906) was drilled in the Aranyos Valley in Veszprém and the Mencshely–1 (Met–1 borehole, N 46°955, E 17°720) is located ∼2 km NE to the village Mencshely. The Zs–14 borehole (N 47° 559, E 18 708) was drilled in the SE foreland of the Gerecse Mountains in the Zsámbék Basin, ∼25 km NW to Budapest.

#### 2.1.1 Palynomorphs from the Veszprém Marl Formation

See Fig. 1, Fig. 2, Fig. 3

### 2.2 Methods

#### 2.2.1 Palynological sampling and laboratory techniques

The preparation procedures include standard palynological processing techniques [4]. Approximately 10 g of sediment were crushed and spiked with a known quantity of *Lycopodium* spores (one tablet/12077 spores) to allow for calculation of palynomorph concentrations followed by acid treatment with HCl (10%), concentrated HF and heavy liquid separation (ZnCl_2_, density 2.9 g/cm^3^). The samples were left in hot concentrated HF (65 °C) in a water bath for two days in order to dissolve the silicate fraction. After washing, the organic residues were sieved to isolate the 250-15 μm size fractions. After the heavy liquid separation, several samples from the Zsámbék–14 borehole were further treated with 10% sodium hypochlorite for 12 hours in order to decrease the high amount of AOM [5]. Unfortunately, the bleaching procedure was not successful and the amount of AOM did not decrease. Slides were glued with Entellan, an epoxy resin based mounting medium. The organic residues are curated at the Department of Geosciences, University of Oslo, Norway. Slides were observed with a standard trinocular Zeiss No. 328883 type microscope connected to an AxioCam ERc5s camera and Zen 2011 software. The organic residues and palynological slides are curated at the Department of Geosciences, University of Oslo. In each sample ∼300 terrestrial palynomorphs (spores and pollen) were counted. After scanning two complete slides the remaining slides were scanned to check for additional taxa. Tables of raw palynomorph counts are available in the supplementary files (S1eS3). The abundance of undetermined palynomorphs, aquatics and *Lycopodium* grains was documented during the quantitative palynological analysis but they were excluded from the palynomorph sum. Pollen diagrams displaying the relative abundance of the palynomorphs was created in Tilia/TiliaGraph computer program. Stratigraphically constrained palynomorph assemblages were determined by cluster analysis (CONISS) built in the Tilia program. The pollen diagrams display only the counted taxa; specimens found after counting and aquatics were excluded from the cluster analysis.

Palynofacies analysis was performed on all samples. The different types of organic matter components are distinguished based on the terminology of Oboh-Ikuenobe & de Villiers [6] (see Table 1). In each sample approximately 300 sedimentary organic particles (SOM) were counted (Supplementary S1eS3).

#### 2.2.2 Ecological signal of the palynomorphs and the SEG method

The ecological interpretation of the dispersed palynomorphs is based on the hygrophytic/xerophytic ratio introduced of Visscher & Van der Zwan ([7]) and the sporomorph ecogroup (SEG) method of Abbink et al. [8]. For details see the original research article Baranyi et al. [1]. The ecological affinity of each spore & pollen type is summarized in Table 2.

#### 2.2.3 Data analysis

Principal component analysis (PCA) was used to reveal the ecological relationship between the dispersed sporomorph types and the presumed parent plants [10]. The PCA routine finds the eigenvalues and eigenvectors in a variance-covariance matrix of the data set. The eigenvalue gives the measure of the variance accounted for by the corresponding components (eigenvector), which is also displayed as the percentages of variance accounted for by each of these components [10]. The principal components are illustrated graphically on two axes as a scatter plot of the data points and variables [10]. The component loadings or species scores on each axis describe the contribution of each of the original variables (e.g., species, taxa) to these environmental trends [11]. Component scores, i.e., sample scores are derived from the component loadings and the original data, so that the highest and lowest scores indicate samples containing the most influential taxa for that axis [11]. When plotted against depth or time, variations in sample score can reveal trends of the ecological/environmental factors represented by the component (axes) in the PCA. The PCA diagram was plotted with PAST.

#### 2.2.4 X-ray fluorescence measurements

Major element analysis was performed by a Philips PW 2404 X-ray fluorescence spectrometer (XRF) with 4 kW Rh-anode, LiF200, PE002-C GE, 111-C, PX-1 analysator crystals, 27/37 mm collimator configuration, scintillator duplex detector at the Department of Earth and Environmental Sciences, University of Pannonia (Veszprém, Hungary). A mass of 1.6 g of selected bulk rock samples (powdered to an average grain size of ∼10 μm) was weighed and mixed with 0.4 g of H_3_BO_3_. The mixture was homogenized using ethanol of analytical purity and pressed under 3000 kg to produce tablets which were measured directly. Total loss on ignition (LOI) was gravimetrically measured after a two-step heating at 105 °C and at 1000 °C, each for 2 hours. The experimental standard deviation ranges 3–6% for each major element measured, but it does 9–10% for Na_2_O.

#### 2.2.5 Weathering indices

The weathering indices were calculated for 108 samples (Supplementary S6). The alpha-indices (α_i_) measure the ratio between the concentration of a mobile element and the concentration of an immobile element with similar magmatic compatibility from the same sediment samples [12] (Supplementary S6).

These elemental ratios are then compared to that in the upper continental crust (UCC [13]). Gaillardet et al. ([12]) used six highly mobile alkali and alkaline earth major elements (Ca, Mg, Sr, Na, K, Ba) as proxies but Ca, Mg and Sr, are usually enriched in the carbonate rocks relative to the UCC and to the average shale. As the investigated rock samples are enriched in clastic material, only Na, K and Ba are selected to calculate α-indices in the present work. As the weathering study targets only the silicate fraction of the rocks, determination of silicate bound fraction of these elements causes hampered analytical procedure and significantly increased chance of a misinterpretation. To avoid effects of element dilution by carbonate compounds and to minimize uncertainties related to the determination of the reference values (i.e. upper continental crust, UCC) and to compositional heterogeneity in lithology of the source area, each mobile element is normalized to the immobile, weathering resistant element aluminium [14]. For each studied mobile element (E) the normalized value is calculated as: α^Al^_E_ = (Al/E)_sample_/(Al/E)_UCC_. The applied weathering index calculations are the following:

(1) α^Al^_Na_ = (Al/Na)_sample_/(Al/Na)_UCC_

(2) α^Al^_K_ = (Al/K)_sample_/(Al/K)_UCC_

(3) α^Al^_Ba_ = (Al/Ba)_sample_/(Al/Ba)_UCC_

The concentration of each element and the calculated α_i_ values are available in the Supplementary S6.
