# Supplementary material for: Palynological and X-ray fluorescence (XRF) data of Carnian (Late Triassic) formations from western Hungary
Source: Data Brief. 2019 Mar 20;23:103858. doi: 10.1016/j.dib.2019.103858 (PMC6661250; doi:10.1016/j.dib.2019.103858)
Supplement: Multimedia component 5 [file mmc5.docx]

# **S4 List of all identified palynomorphs**

This list includes all valid palynomorph taxa below generic level from the Carnian successions of the Transdanubian Range, with full author citations. The palynomorphs are listed alphabetically within nine groups.

3.1 Spores

*Anapiculatisporites telephorus* (Pautsch 1958) Klaus 1960

*Aratrisporites granulatus* (Klaus 1960) Playford & Dettmann 1965

*Aratrisporites palettae* (Klaus 1960) Schulz 1967

*Aratrisporites paraspinosus* Klaus 1960

*Aratrisporites scabratus* Klaus 1960

*Baculatisporites* sp.

*Calamospora tener* (Leschik 1955) De Jersey 1962

*Camarazonosporites rudis* (Leschik 1956) Klaus 1960

*Conbaculatisporites mesozoicus* Klaus 1960

*Concavisporites toralis* (Leschik 1955) Nilsson 1958

*Converrucosiporites tumolosus* (Leschik 1956) Roghi 2004

*Cyclogranisporites* sp.

*Cyclotriletes margaritatus* Mädler 1964

*Deltoidospora* sp.

*Dictyophyllidites harrisii* Couper 1958

*Gibeosporites lativerrucosus* Leschik 1959 synonym: Apiculatasporites lativerrucosus Leschik 1955

*Gordonispora fossulata* Van der Eem 1983

*Kraeuselisporites cooksonae* (Klaus 1960) Dettmann 1963

*Kyrtomisporis erveii* Van der Eem 1983

*Laevigatisporites robostus* Leschik 1956

cf. *Leschikisporites aduncus* (Leschik 1956) R. Potonié 1958

*Lycopodiacidites kuepperi* Klaus 1960

*Lycopodiacidites* sp.

*Osmundacidites wellmannii* Couper 1953

*Neoraistrickia taylorii* Playford et Dettmann 1965

*Paraconcavisporites lunzensis* Klaus 1960

*Porcellispora longdonensis* (Clarke 1965) Scheuring 1970 emend. Morbey 1975

*Reticulatisporites dolomiticus* Blendinger 1988

*Striatella seebergensis* Mädler 1964 synonyms: *Corrugatisporites klausi* Kavary 1972, *Duplexisporites* *gyratus* (Playford & Dettmann); de Jersey & Paten 1964; *Asseretospora gyrata* (Playford & Dettmann) Schuurman 1977

*Todisporites major* Couper 1958

*Todisporites rotundiformis* (Maljavkina 1943) Pocock 1970

*Uvaesporites gadensis* Praehauser-Enzenberg 1970

*Verrucosisporites morulae* Klaus 1960

*Zebrasporites fimbriatus* Klaus 1960

3.2 Bisaccate pollen grains

*Alisporites aequalis* Mädler 1964

*Alisporites giganteus* (Danzé-Corsin & Laveine 1963) Cornet 1977

*Alisporites robostus* Nilsson 1958

*Alisporites toralis* (Leschik 1956) Clarke 1965

*Alisporites* sp.

*Brachysaccus neomundanus* (Leschik 1956) Mädler 1964

*Ellipsovelatisporites plicatus* Klaus 1960

*Infernopollenites parvus* Scheuring 1970

*Infernopollenites sulcatus* (Pautsch 1958) Scheuring 1970

*Lueckisporites singhii* Balme 1970

*Lunatisporites acutus* (Leschik 1955) Scheuring 1970

*Microcachryidites doubingeri* Klaus 1964

*Ovalipollis brutus* Scheuring 1970

*Ovalipollis* *lunzensis* Klaus 1960

*Ovalipollis* *minimus* Scheuring 1970

*Ovalipollis* *ovalis* Krutzsch 1955

*Ovalipollis* *septimus* Scheuring 1970

*Parillinites* sp.

*Pityosporites* *devolvens* Leschik 1956

*Platysaccus* *queenslandi* de Jersey 1962

*Protodiploxypinus* *fastidiosus* (Jansonius 1962) Warrington 1974

*Protodiploxypinus* *gracilis* Scheuring 1970

*Staurosaccites* *quadrifidus* Dolby 1976

*Striatoabietites* *aytugii* (Visscher 1966) Scheuring 1970

*Sulcatisporites* *krauseli* Mädler 1964

*Triadispora* *bölchi* Scheuring 1970

*Triadispora* *crassa* Klaus 1964

*Triadispora* *epigona* Scheuring 1970

*Triadispora* *obscura* Scheuring 1970

*Triadispora* *plicata* Klaus 1964

*Triadispora* *stabilis* (Scheuring 1970) Scheuring 1978

*Triadispora sulca*ta Scheuring 1970

*Triadispora suspecta* Scheuring 1970

3.3 Monosaccate pollen grains

*Enzonalasporites tenuis* Leschik 1956

*Enzonalasporites vigens* (Leschik 1956) Scheuring 1970

*Patinasporites densus* (Leschik 1956) Scheuring 1970

*Patinasporites explanatus* (Leschik 1956) Góczán & Oravecz-Scheffer 1996b synonym: *Accinctisporites* in Roghi 2004

*Patinasporites iustus* Klaus 1960

*Patinasporites toralis* Scheuring 1970

*Pseudoenzonalasporites summus* Scheuring 1970

*Vallasporites ignacii* Leschik 1956

3.4 Circumpolles pollen grains

*Camerosporites secatus* (Leschik 1955) Scheuring 1970

*Duplicisporites* continuus Praehauser-Enzenberg 1970

*Duplicisporites* *granulatus* Leschik 1956

*Duplicisporites* *mancus* Klaus 1960

cf. *Partitisporites* *tenebrosus* (Scheuring 1970) Van der Eem 1983

*Partitisporites* *maljawkinae* (Klaus 1960) Van der Eem 1983

*Partitisporites* *novimundanus* Leschik 1956

*Partitisporites* *scurrilis* (Scheuring 1970) Van der Eem 1983

*Praecirculina* *granifer* (Leschik 1956) Klaus 1960

*Partitisporites* sp. indet.

3.5 Non-saccate pollen grains

*Aulisporites astigmosus* (Leschik 1955) Klaus 1960

*Laricoidites* sp.

*Equisetosporites* *chinleana* (Daugherty 1941) Scott 1960

*Cycadopites* sp.

*Lagenella* *martinii* Klaus 1960

3.6 Incertae sedis

*Brodispora striata* Clarke 1965

3.7 Aquatic palynomorphs

*Baltisphaeridium* sp.

*Botryococcus braunii* Kützing 1849

*Dictyotidium reticulatum* Schulz 1965

Dinocyst indet.

*Heibergella* sp.

*Leiofusa* sp.

*Leiosphaeridia* sp.

*Micrhystridium* sp. 1 (short, thin processes)

*Micrhystridium* sp. 2 (long processes, with broad base)

*Micrhystridium* sp. 3 (long, thin processes)

*Schizosporis* sp.

*Tasmanites* sp.

*Veryhachium* sp.

**Acknowledgements**

WMK and VB acknowledge funding from the Faculty of Mathematics and Natural Sciences at the University of Oslo (Norway). Mufak Said Naoroz (UiO) is thanked for his help in the processing of the palynological samples. We thankfully acknowledge the help of János Haas on a fieldtrip in the Transdanubian Range. Steven Mueller helped with the sampling.
